# Supplementary material for: The Effect of a Combined Gluten- and Casein-Free Diet on Children and Adolescents with Autism Spectrum Disorders: A Systematic Review and Meta-Analysis
Source: Nutrients. 2021 Jan 30;13(2):470. doi: 10.3390/nu13020470 (PMC7912271; doi:10.3390/nu13020470)
Supplement: Supplementary file 1 [file nutrients-13-00470-s001.pdf]

Amélie Keller, Marie Louise Rimestad, Jeanett Friis Rohde, Birgitte Holm Petersen, Christoffer Bruun Korfitsen, Simon Tarp, Marlene Briciet Lauritsen, Mina Nicole Händel

Supplementary material

Contents

PRISMA checklist 2
Search description 5
Search for systematic reviews/meta-analyses 5
Medline (030120) 5
Embase (030120) 7
Cinahl (030120) 9
Primary search 10
Medline (270120) 10
Embase (270120) 12
Cinahl (270120) 14
Assessment of the methodological quality of the included systematic reviews (AMSTAR). 15
Characteristics of excluded studies 16

PRISMA checklist

Section/topic	#	Checklist item	Reported on page #
**TITLE**			
Title	1	Identify the report as a systematic review, meta-analysis, or both.	1
**ABSTRACT**			
Structured summary	2	Provide a structured summary including, as applicable: background; objectives; data sources; study eligibility criteria, participants, and interventions; study appraisal and synthesis methods; results; limitations; conclusions and implications of key findings; systematic review registration number.	2
**INTRODUCTION**			
Rationale	3	Describe the rationale for the review in the context of what is already known.	3-4
Objectives	4	Provide an explicit statement of questions being addressed with reference to participants, interventions, comparisons, outcomes, and study design (PICOS).	4
**METHODS**			
Protocol and registration	5	Indicate if a review protocol exists, if and where it can be accessed (e.g., Web address), and, if available, provide registration information including registration number.	5
Eligibility criteria	6	Specify study characteristics (e.g., PICOS, length of follow-up) and report characteristics (e.g., years considered, language, publication status) used as criteria for eligibility, giving rationale.	5-6
Information sources	7	Describe all information sources (e.g., databases with dates of coverage, contact with study authors to identify additional studies) in the search and date last searched.	5
Search	8	Present full electronic search strategy for at least one database, including any limits used, such that it could be repeated.	5
Study selection	9	State the process for selecting studies (i.e., screening, eligibility, included in systematic review, and, if applicable, included in the meta-analysis).	5-6
Data collection process	10	Describe method of data extraction from reports (e.g., piloted forms, independently, in duplicate) and any processes for obtaining and confirming data from investigators.	7
Data items	11	List and define all variables for which data were sought (e.g., PICOS, funding sources) and any assumptions and simplifications made.	7

Risk of bias in individual studies	12	Describe methods used for assessing risk of bias of individual studies (including specification of whether this was done at the study or outcome level), and how this information is to be used in any data synthesis.	8
Summary measures	13	State the principal summary measures (e.g., risk ratio, difference in means).	9
Synthesis of results	14	Describe the methods of handling data and combining results of studies, if done, including measures of consistency (e.g., I^2) for each meta-analysis.	9

Section/topic	#	Checklist item	Reported on page #
Risk of bias across studies	15	Specify any assessment of risk of bias that may affect the cumulative evidence (e.g., publication bias, selective reporting within studies).	8
Additional analyses	16	Describe methods of additional analyses (e.g., sensitivity or subgroup analyses, meta-regression), if done, indicating which were pre-specified.	NA
**RESULTS**			
Study selection	17	Give numbers of studies screened, assessed for eligibility, and included in the review, with reasons for exclusions at each stage, ideally with a flow diagram.	10-12
Study characteristics	18	For each study, present characteristics for which data were extracted (e.g., study size, PICOS, follow-up period) and provide the citations.	12, table 1
Risk of bias within studies	19	Present data on risk of bias of each study and, if available, any outcome level assessment (see item 12).	19, Fig 4-9
Results of individual studies	20	For all outcomes considered (benefits or harms), present, for each study: (a) simple summary data for each intervention group (b) effect estimates and confidence intervals, ideally with a forest plot.	16-19, Fig 4-9
Synthesis of results	21	Present results of each meta-analysis done, including confidence intervals and measures of consistency.	16-19
Risk of bias across studies	22	Present results of any assessment of risk of bias across studies (see Item 15).	19-20, Fig 4-9, supplemental material
Additional analysis	23	Give results of additional analyses, if done (e.g., sensitivity or subgroup analyses, meta-regression [see Item 16]).	NA
**DISCUSSION**			

Summary of evidence	24	Summarize the main findings including the strength of evidence for each main outcome; consider their relevance to key groups (e.g., healthcare providers, users, and policy makers).	23
Limitations	25	Discuss limitations at study and outcome level (e.g., risk of bias), and at review-level (e.g., incomplete retrieval of identified research, reporting bias).	24-26
Conclusions	26	Provide a general interpretation of the results in the context of other evidence, and implications for future research.	26
**FUNDING**			
Funding	27	Describe sources of funding for the systematic review and other support (e.g., supply of data); role of funders for the systematic review.	27

From: Moher D, Liberati A, Tetzlaff J, Altman DG, The PRISMA Group (2009). Preferred Reporting Items for Systematic Reviews and Meta-Analyses: The PRISMA Statement. PLoS Med 6(7): e1000097. doi:10.1371/journal.pmed1000097

For more information, visit: **www.prisma-statement.org**.

Search description

Search for systematic reviews/meta-analyses

Medline (030120)

Database(s): **Ovid MEDLINE(R) and Epub Ahead of Print, In-Process & Other Non-Indexed Citations, Daily and Versions(R)** 1946 to January 02, 2020
Search Strategy:

#	Searches	Results
1	(autism* or ASD).mp. [mp=title, abstract, original title, name of substance word, subject heading word, floating sub-heading word, keyword heading word, organism supplementary concept word, protocol supplementary concept word, rare disease supplementary concept word, unique identifier, synonyms]	49062
2	Autism Spectrum Disorder*.mp. or Autism Spectrum Disorder/	23277
3	Autism Disorder*.mp.	119
4	Autistic Disorder/ or Autistic Disorder*.mp.	20451
5	autistic*.mp.	24796
6	Asperger Syndrome/ or asperger*.mp.	2724
7	asperger*.mp.	2724
8	Neurodevelopmental Disorders/ or neurodevelopmental disorder*.mp.	10279
9	Child Development Disorders, Pervasive.mp. or Child Development Disorders, Pervasive/	6531
10	or/1-9	63127
11	(((systematic or method* or rapid or integrative or umbrella) adj3 (review* or overview* or study or studies or search* or approach*)) or meta analy* or meta-analy* or metaanaly*).ti,ab,kw,kf,bt.	785685
12	(pooled adj1 (data or analys*)).ti,ab.	17385
13	(pubmed or medline or embase or cochrane or "web of science" or psycinfo or psychinfo or scopus).ti,ab.	220226
14	Cochrane.jw.	14875
15	Diet, Gluten-Free/ or gluten*.mp. or Glutens/	14188

16	casein*.mp.	34829
17	Celiac Disease/ or Celiac Disease*.mp.	22682
18	Caseins/ or Food Hypersensitivity/ or casein intolerance*.mp. or Milk Proteins/	40649
19	gluten intolerance*.mp.	352
20	casein-free*.mp.	105
21	casein intolerance*.mp.	1
22	Food Hypersensitivit*.mp.	15847
23	(diet* and elimination*).mp. [mp=title, abstract, original title, name of substance word, subject heading word, floating sub-heading word, keyword heading word, organism supplementary concept word, protocol supplementary concept word, rare disease supplementary concept word, unique identifier, synonyms]	5999
24	elimination diet*.mp.	1104
25	(elimination* and casein*).mp. [mp=title, abstract, original title, name of substance word, subject heading word, floating sub-heading word, keyword heading word, organism supplementary concept word, protocol supplementary concept word, rare disease supplementary concept word, unique identifier, synonyms]	211
26	(elimination* and gluten*).mp. [mp=title, abstract, original title, name of substance word, subject heading word, floating sub-heading word, keyword heading word, organism supplementary concept word, protocol supplementary concept word, rare disease supplementary concept word, unique identifier, synonyms]	204
27	GI.mp. or Gastrointestinal Diseases/	78781
28	gastrointestinal*.mp.	360425
29	15 or 16 or 17 or 18 or 19 or 20 or 21 or 22 or 23 or 24 or 25 or 26 or 27 or 28	468270
30	11 or 12 or 13 or 14	868513
31	10 and 29 and 30	99

Embase (030120)

Database(s): **Embase** 1996 to 2019 Week 52
Search Strategy:

#	Searches	Results
1	(autism* or ASD).mp. [mp=title, abstract, heading word, drug trade name, original title, device manufacturer, drug manufacturer, device trade name, keyword, floating subheading word, candidate term word]	75915
2	Autism Spectrum Disorder*.mp. or Autism Spectrum Disorder/	50988
3	Autism Disorder*.mp.	186
4	Autistic Disorder/ or Autistic Disorder*.mp.	20572
5	autistic*.mp.	13831
6	Asperger Syndrome/ or asperger*.mp.	5238
7	asperger*.mp.	5238
8	Neurodevelopmental Disorders/ or neurodevelopmental disorder*.mp.	141599
9	Child Development Disorders, Pervasive.mp. or Child Development Disorders, Pervasive/	19084
10	or/1-9	211156
11	(((systematic or method* or rapid or integrative or umbrella) adj3 (review* or overview* or study or studies or search* or approach*)) or meta analy* or meta-analy* or metaanaly*).ti,ab,kw.	1018589
12	(pooled adj1 (data or analys*)).ti,ab.	26200
13	(pubmed or medline or embase or cochrane or "web of science" or psycinfo or psychinfo or scopus).ti,ab.	268639
14	Cochrane.jw.	21196
15	Diet, Gluten-Free/ or gluten*.mp. or Glutens/	17028
16	casein*.mp.	26458
17	Celiac Disease/ or Celiac Disease*.mp.	26118
18	Caseins/ or Food Hypersensitivity/ or casein intolerance*.mp. or Milk Proteins/	23477

19	gluten intolerance*.mp.	395
20	casein-free*.mp.	147
21	casein intolerance*.mp.	3
22	Food Hypersensitivit*.mp.	1454
23	(diet* and elimination*).mp. [mp=title, abstract, heading word, drug trade name, original title, device manufacturer, drug manufacturer, device trade name, keyword, floating subheading word, candidate term word]	7884
24	elimination diet*.mp.	1962
25	(elimination* and casein*).mp. [mp=title, abstract, heading word, drug trade name, original title, device manufacturer, drug manufacturer, device trade name, keyword, floating subheading word, candidate term word]	262
26	(elimination* and gluten*).mp. [mp=title, abstract, heading word, drug trade name, original title, device manufacturer, drug manufacturer, device trade name, keyword, floating subheading word, candidate term word]	314
27	GI.mp. or Gastrointestinal Diseases/	76399
28	gastrointestinal*.mp.	461540
29	15 or 16 or 17 or 18 or 19 or 20 or 21 or 22 or 23 or 24 or 25 or 26 or 27 or 28	561687
30	11 or 12 or 13 or 14	1126220
31	10 and 29 and 30	594

Cinahl (030120)

#	Query	Results
S19	S16 AND S17 AND S18	33
S18	S5 OR S6 OR S7 OR S8 OR S9 OR S10 OR S11 OR S12 OR S13	224,438
S17	S1 OR S2	28,070
S16	S14 OR S15	148,809
S15	(MH "Meta Analysis") OR "meta analys*"	72,478
S14	(MH "Systematic Review") OR "systematic review*"	119,577
S13	(MH "Food Hypersensitivity+") OR "food hypersensitivities"	5,627
S12	food hypersensitivit*	5,151
S11	elimination diet*	236
S10	gluten* and elimination*	55
S9	casein* and elimination*	19
S8	diet* and elimination*	767
S7	(MH "Diet+") OR (MH "Restricted Diet+") OR "diet*"	219,839
S6	casein*	1,383
S5	(MH "Caseins")	685
S4	gluten*	4,020
S3	(MH "Celiac Disease+") OR (MH "Gluten") OR "gluten"	6,214
S2	(MH "Autistic Disorder") OR (MH "Asperger Syndrome") OR "autism"	26,521
S1	autism* or ASD	24,702

Primary search

Medline (270120)

Database(s): **Ovid MEDLINE(R) and Epub Ahead of Print, In-Process & Other Non-Indexed Citations, Daily and Versions(R)** 1946 to January 24, 2020
Search Strategy:

#	Searches	Results
1	(autism* or ASD).mp. [mp=title, abstract, original title, name of substance word, subject heading word, floating sub-heading word, keyword heading word, organism supplementary concept word, protocol supplementary concept word, rare disease supplementary concept word, unique identifier, synonyms]	49444
2	Autism Spectrum Disorder*.mp. or Autism Spectrum Disorder/	23524
3	Autism Disorder*.mp.	119
4	Autistic Disorder/ or Autistic Disorder*.mp.	20488
5	autistic*.mp.	24872
6	Asperger Syndrome/ or asperger*.mp.	2730
7	asperger*.mp.	2730
8	Neurodevelopmental Disorders/ or neurodevelopmental disorder*.mp.	10391
9	Child Development Disorders, Pervasive.mp. or Child Development Disorders, Pervasive/	6532
10	or/1-9	63595
11	Diet, Gluten-Free/ or gluten*.mp. or Glutens/	14243
12	casein*.mp.	34896
13	Celiac Disease/ or Celiac Disease*.mp.	22734
14	Caseins/ or Food Hypersensitivity/ or casein intolerance*.mp. or Milk Proteins/	40759
15	gluten intolerance*.mp.	354
16	casein-free*.mp.	105
17	casein intolerance*.mp.	1
18	Food Hypersensitivit*.mp.	15894

19	(diet* and elimination*).mp. [mp=title, abstract, original title, name of substance word, subject heading word, floating sub-heading word, keyword heading word, organism supplementary concept word, protocol supplementary concept word, rare disease supplementary concept word, unique identifier, synonyms]	6014
20	elimination diet*.mp.	1108
21	(elimination* and casein*).mp. [mp=title, abstract, original title, name of substance word, subject heading word, floating sub-heading word, keyword heading word, organism supplementary concept word, protocol supplementary concept word, rare disease supplementary concept word, unique identifier, synonyms]	211
22	(elimination* and gluten*).mp. [mp=title, abstract, original title, name of substance word, subject heading word, floating sub-heading word, keyword heading word, organism supplementary concept word, protocol supplementary concept word, rare disease supplementary concept word, unique identifier, synonyms]	204
23	GI.mp. or Gastrointestinal Diseases/	79027
24	gastrointestinal*.mp.	361778
25	11 or 12 or 13 or 14 or 15 or 16 or 17 or 18 or 19 or 20 or 21 or 22 or 23 or 24	469898
26	10 and 25	1133
27	limit 26 to (controlled clinical trial or randomized controlled trial)	36
28	(((random* or cluster-random* or control?ed or crossover or cross-over or blind* or mask*) adj4 (trial*1 or study or studies or analy*)) or rct).ti,ab,kw,kf.	675999
29	(placebo* or single-blind* or double-blind* or triple-blind*).ti,ab.	276548
30	((single or double or triple) adj2 (blind* or mask*)).ti,ab.	169913
31	((patient* or person* or participant* or population* or allocate* or assign*) adj3 (random* or blind* or mask*)).ti,ab,kw,kf.	251574
32	28 or 29 or 30 or 31	873153
33	26 and 32	103
34	27 or 33	109

35	limit 34 to yr="2016 - 2020"	44

Embase (270120)

Database(s): **Embase** 1996 to 2020 Week 04
Search Strategy:

#	Searches	Results
1	(autism* or ASD).mp. [mp=title, abstract, heading word, drug trade name, original title, device manufacturer, drug manufacturer, device trade name, keyword, floating subheading word, candidate term word]	76615
2	Autism Spectrum Disorder*.mp. or Autism Spectrum Disorder/	51576
3	Autism Disorder*.mp.	189
4	Autistic Disorder/ or Autistic Disorder*.mp.	21123
5	autistic*.mp.	13934
6	Asperger Syndrome/ or asperger*.mp.	5258
7	asperger*.mp.	5258
8	Neurodevelopmental Disorders/ or neurodevelopmental disorder*.mp.	143116
9	Child Development Disorders, Pervasive.mp. or Child Development Disorders, Pervasive/	19000
10	or/1-9	213283
11	Diet, Gluten-Free/ or gluten*.mp. or Glutens/	17164
12	casein*.mp.	26618
13	Celiac Disease/ or Celiac Disease*.mp.	26301
14	Caseins/ or Food Hypersensitivity/ or casein intolerance*.mp. or Milk Proteins/	23737
15	gluten intolerance*.mp.	400
16	casein-free*.mp.	149
17	casein intolerance*.mp.	3

18	Food Hypersensitivit*.mp.	1455
19	(diet* and elimination*).mp. [mp=title, abstract, heading word, drug trade name, original title, device manufacturer, drug manufacturer, device trade name, keyword, floating subheading word, candidate term word]	7947
20	elimination diet*.mp.	1971
21	(elimination* and casein*).mp. [mp=title, abstract, heading word, drug trade name, original title, device manufacturer, drug manufacturer, device trade name, keyword, floating subheading word, candidate term word]	263
22	(elimination* and gluten*).mp. [mp=title, abstract, heading word, drug trade name, original title, device manufacturer, drug manufacturer, device trade name, keyword, floating subheading word, candidate term word]	314
23	GI.mp. or Gastrointestinal Diseases/	77373
24	gastrointestinal*.mp.	465118
25	11 or 12 or 13 or 14 or 15 or 16 or 17 or 18 or 19 or 20 or 21 or 22 or 23 or 24	566078
26	10 and 25	6352
27	limit 26 to (controlled clinical trial or randomized controlled trial)	678
28	(((random* or cluster-random* or control?ed or crossover or cross-over or blind* or mask*) adj4 (trial*1 or study or studies or analy*)) or rct).ti,ab,kw.	849033
29	(placebo* or single-blind* or double-blind* or triple-blind*).ti,ab.	319592
30	((single or double or triple) adj2 (blind* or mask*)).ti,ab.	185601
31	((patient* or person* or participant* or population* or allocate* or assign*) adj3 (random* or blind* or mask*)).ti,ab,kw.	317765
32	28 or 29 or 30 or 31	1076453
33	26 and 32	966
34	27 or 33	1125
35	limit 34 to yr="2016 - 2020"	241

36	limit 26 to ((randomized controlled trial or controlled clinical trial) and yr="2016 - 2020")	137
37	35 or 36	241

Cinahl (270120)

#	Query	Results
S20	S13 AND S18<br>Limiters - Published Date: 20160101-20191231	39
S19	S13 AND S18	87
S18	S14 OR S15 OR S16 OR S17	473,650
S17	PT Randomized Controlled Trial	86,203
S16	((patient* or person* or participant* or population* or allocate* or assign*) N3 (random* or blind* or mask*))	305,012
S15	(placebo* or single-blind* or double-blind* or triple-blind* or ((single or double or triple) N1 (blind* or mask*))	99,667
S14	(((random* or cluster-random* or control#ed or crossover or cross-over or blind* or mask*) N3 (trial* or study or studies or analy*)) or rct)	449,145
S13	S11 AND S12	723
S12	S3 OR S4 OR S5 OR S6 OR S7 OR S8 OR S9 OR S10	228,081
S11	S1 OR S2	28,233
S10	elimination diet*	239
S9	gluten* and elimination*	56
S8	casein* and elimination*	19
S7	diet* and elimination*	772
S6	(MH "Food Hypersensitivity+") OR "food hypersensitivit*"	5,664
S5	(MH "Diet+") OR (MH "Diet, Gluten-Free") OR "diet*"	220,902
S4	(MH "Caseins") OR "casein*"	1,390
S3	(MH "Gluten") OR (MH "Diet, Gluten-Free") OR (MH "Celiac Disease+") OR "gluten*"	6,243
S2	(MH "Autistic Disorder") OR (MH "Asperger Syndrome") OR "autism"	26,670
S1	autism* or ASD	24,852

Assessment of the methodological quality of the included systematic reviews (AMSTAR).

The different domains are presented in the top row. The individual studies are shown in the left column

Citation	List relevant outcomes	Was meta-analysis performed for the relevant outcomes?	1. Was an 'a priori' design provided?	2. Was there duplicate study selection and data extraction?	3. Was a comprehensive literature search performed?	4. Was the status of publication (i.e. grey literature) used as an inclusion	5. Was a list of studies (included and excluded) provided?	6. Were the characteristics of the included studies provided?	7. Was the scientific quality of the included studies assessed and documented?	8. Was the scientific quality of the included studies used appropriately in	9. Were the methods used to combine the findings of studies appropriate?	10. Was the likelihood of publication bias assessed?	11. Was the conflict of interest included?
Piwowarczyk, 2018	NA	NA	No	Yes	Yes	No	Yes	Yes	Yes	No	NA	No	Yes
Elder 2008	NA	NA	No	Yes	Yes	No	Yes	Yes	No	Yes	NA	No	No
Brondino 2015	NA	NA	No	No	Yes	Can't answer	No	Yes	Can't answer	Yes	NA	No	No

Characteristics of excluded studies

Study reference	Reasons for exclusion
Adams, James B.; Audhya, Tapan; Geis, Elizabeth; Gehn, Eva; Fimbres, Valeria; Pollard, Elena L.; Mitchell, Jessica; Ingram, Julie; Hellmers, Robert; Laake, Dana; Matthews, Julie S.; Li, Kefeng; Naviaux, Jane C.; Naviaux, Robert K.; Adams, Rebecca L.; Coleman, Devon M.; Quig, David W.. Comprehensive Nutritional and Dietary Intervention for Autism Spectrum Disorder-A Randomized, Controlled 12-Month Trial. Nutrients 2018;10(3). [DOI: https://dx.doi.org/10.3390/nu10030369]	Wrong intervention
Ghalichi, Faezeh; Ghaemmaghami, Jamal; Malek, Ayyoub; Ostadrahimi, Alireza. Effect of gluten free diet on gastrointestinal and behavioral indices for children with autism spectrum disorders: a randomized clinical trial. World journal of pediatrics : WJP 2016;12(4):436-442. [DOI:]	Wrong intervention
Hyman, S. L.; Stewart, P. A.; Foley, J.; Cain, U.; Peck, R.; Morris, D. D.; Wang, H.; Smith, T.. The Gluten-Free/Casein-Free Diet: A Double-Blind Challenge Trial in Children with Autism. Journal of Autism and Developmental Disorders 2016;46(1):205-220. [DOI: 10.1007/s10803-015-2564-9 [doi]]	Wrong intervention
Hyman, Susan L.; Stewart, Patricia A.; Foley, Jennifer; Cain, Usa; Peck, Robin; Morris, Danielle D.; Wang, Hongyue; Smith, Tristram. The Gluten-Free/Casein-Free Diet: A Double-Blind Challenge Trial in Children with Autism. Journal of Autism and Developmental Disorders 2016;46(1):205-220. [DOI: https://dx.doi.org/10.1007/s10803-015-2564-9]	Wrong intervention
Mari-Bauset, Salvador; Llopis-Gonzalez, Agustin; Zazpe, Itziar; Mari-Sanchis, Amelia; Suarez-Varela, Maria. Nutritional Impact of a Gluten-Free Casein-Free Diet in Children with Autism Spectrum Disorder. Journal of Autism and Developmental Disorders 2016;46(2):673-84. [DOI: https://dx.doi.org/10.1007/s10803-015-2582-7]	Wrong intervention

Pusponegoro, H. D.; Ismael, S.; Firmansyah, A.; Sastroasmoro, S.; Vandenplas, Y.. Gluten and casein supplementation does not increase symptoms in children with autism spectrum disorder. Acta Paediatrica (Oslo, Norway : 1992) 2015;104(11):e500-5. [DOI: 10.1111/apa.13108 [doi]]	Wrong intervention
Seung, H.; Rogalski, Y.; Shankar, M.; Elder, J.. Seung H, Rogalski Y, Shankar M, Elder J. The gluten- and casein-free diet and autism: communication outcomes from a preliminary double-blind clinical trial.. 2007;15(4):337-345. [DOI:]	Wrong intervention

The effect of a combined gluten- and casein-free diet on children and adolescents with autism spectrum disorders: A systematic review and meta-analysis

Amélie Keller, Marie Louise Rimestad, Jeanett Friis Rohde, Birgitte Holm Petersen, Christoffer Bruun Korfitsen, Simon Tarp, Marlene Briciet Lauritsen, Mina Nicole Händel
